# ACE I/D Polymorphism in Hypertensive Patients of Kashmiri Population

**DOI:** 10.4021/cr101e

**Published:** 2010-11-20

**Authors:** A. Syed Sameer, Nidda Syeed, Shahid A. Tak, Samina Bashir, Saniya Nissar, Mushtaq A. Siddiqi

**Affiliations:** a Departments of Immunology and Molecular Medicine, Sher-I-Kashmir Institute of Medical Sciences, Soura, Srinagar, Kashmir, 190011, India; b Departments of Clinical Biochemistry, Sher-I-Kashmir Institute of Medical Sciences, Soura, Srinagar, Kashmir, India; c Departments of Cardiology, Sher-I-Kashmir Institute of Medical Sciences, Soura, Srinagar, Kashmir, India; d Department of Clinical Biochemistry, Kashmri University, Hazratbal, Srinagar, Kashmir, India

**Keywords:** Hypertension, ACE, Polymorphism, RFLP, Kashmir

## Abstract

**Background:**

The angiotensin-converting enzyme (ACE) gene in humans has an insertion-deletion (I/D) polymorphic state in intron 16 on chromosome 17q23. This polymorphism has been widely investigated in different diseases. In this study we aimed to investigate the ACE I/D genotype frequency in hypertensive cases in Kashmiri population.

**Materials and Methods:**

We designed a case control study, where 52 hypertensive cases were studied for ACE I/D polymorphism against 150 age/sex matched controls taken from general population. The polymorphisms of ACE gene were investigated using polymerase chain reaction for detection of ACE I/D genotype. Fisher’s Chi square test was used for calculation of P value and OR.

**Results:**

We found the frequency of ACE DD genotype to be 46.15% (24/52), II 23.07% (12/52) and DI 30.77% (16/52) in 52 hypertensive cases.

**Conclusions:**

The ACE I/D genotype is positively associated with hypertension in our population.

## Introduction

Hypertension (HT, HTN or HPN) is a chronic medical condition in which the blood pressure is elevated [1]. Hypertension is categorized into essential (primary) representing 90-95% of cases and secondary which occurs as a result of other conditions, such as kidney disease or tumors (adrenal adenoma or pheochromocytoma) [2]. The prevalence of hypertension in India varies from as less as 30% to as much as 61% [3-8]. The Sentinel Surveillance Project (WHO) [9] documented 28% overall prevalence of hypertension (criteria: = JNC VI) from 10 regions of the country in the age group of 20 - 69. The scenario in Kashmir is not different from the reported literature from other parts of the India, because of the two decade long turmoil raging in all corners of the valley. Based on the medical registry of Sher-i-Kashmir Institute of Medical Sciences (SKIMS), hypertension in Kashmir is found in almost 60% of the population.

Angiotensin-converting enzyme (ACE), a key zinc metalloenzyme of the rennin-angiotensin system is widely distributed in body in the kidney [10]. The ACE catalyzes the conversion of angiotensin I to the biologically active peptide, angiotensin II, which is involved in the control of fluid-electrolyte balance and systemic blood pressure [11].

The ACE gene is located on long arm of chromosome 17 (17q23.3). The gene is 21 kilo bases (kb) long and comprises 26 exons and 25 introns. More than 160 ACE gene polymorphisms have been reported so far and most of which are single nucleotide polymorphisms (SNPs). Only 34 of those polymorphisms are located in coding region of this gene [12]. Rigat et al (1990) was the first to report the insertion/deletion (I/D) polymorphism of ACE [13]. This polymorphism is characterized by the presence (insertion) or absence (deletion) of a 287 bp *Alu*Ya5 element inside intron 16 producing three genotypes (II homozygote, ID heterozygote and DD homozygote). Although I/D polymorphism is located in a non-coding region (namely intron) of the ACE gene, several investigators have found that the D allele is related to increased activity of ACE in serum. The highest serum ACE activity was seen in the DD genotype while the lowest was seen in the II genotype [14]. The number of studies carried out around the world suggested the genetic predisposition of the ACE I/D polymorphism with several diseases including coronary heart diseases, stroke, hypertension and diabetes mellitus [15-18]. However, conflicting results have been reported regarding the association between ACE polymorphism and disease [19, 20]. Moreover, various reports were published suggesting inter-ethnic variations in the frequency of allelic forms of the ACE genes [21, 22].

Angiotensin II is an aldosterone-stimulating peptide with a direct, potent vasopressive effect on the peripheral vasculature, and plays a pivotal role in electrolyte and circulatory homeostasis. It is converted from its precursor, angiotensin I, by the catalytic action of the dipeptidylcarboxypeptidase-ACE [23]. As the ACE I/D polymorphism is partially associated with the plasma ACE level [24], the ACE DD genotype increases the plasma ACE concentration and the risk for numerous cardiovascular-renal diseased states, such as myocardial infarction, cardiomyopathy, IgA nephropathy, and diabetic nephropathy. The findings from case-control studies have not been consistently positive [25-29].

Therefore, we carried out a case-control study in our population to determine if this ACE I/D polymorphism is associated with an altered risk of hypertension in our population.

## Materials and Methods

### Study population

This study included 52 hypertensive and cardiac patients. All patients were recruited from Department of Cardiology of this Institute from March 2009 to February 2010. Hypertension was defined by the use of one or more antihypertensive medications and/or a blood pressure not less than 140 mm Hg systolic or 90 mm Hg diastolic. Blood samples of 150 age and sex matched cases with no signs of any cardiac disease were collected to serve as external controls.

Data on all CRC patients were obtained from personal interviews with patients and/or guardians and medical records. The clinical characteristics of the patients at the time of diagnosis, including age, gender, dwelling, stress level, office blood pressure, urinary protein excretion (g/d), serum creatinine level (sCr, mg/dL), and 24-hour creatinine clearance (CCr, mL/min) were retrospectively investigated. Echocardiographic analysis of all hypertensive cases was carried out to know the severity of aortic valve calcification. All patients and/or guardians were informed about the study and their will to participate in this study was taken on predesigned questionnaire (available on request). The collection and use of blood samples (from patients and controls) for this study were previously approved by the appropriate Institutional Ethics Committee.

### DNA extraction and polymerase chain reaction

DNA extraction was performed using ammonium acetate precipitation method. Polymorphism in intron 16 of the ACE gene was assessed by polymerase chain reaction (PCR) under conditions that have been previously described [10].

**Figure 1:**
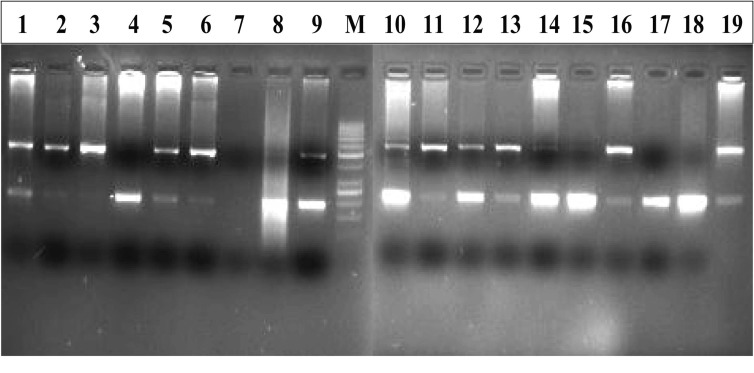
Representative gel picture of ACE DI polymorphism by differential amplification of intron 16 of the ACE gene. Lane M: 50bp ladder; Lanes 1, 2, 5, 6, 9, 10, 11, 12, 13, 14, 16 and 19 show DI form; Lanes 4, 8, 15, 17, 18 and 12 show DD form; Lane 3 shows II form.

The specific segment of ACE gene was amplified by using previously reported primers (Table 1) [10], which amplified 190 bp amplicon in case of homozygous DD genotype, 490 bp in case of homozygous II genotype and both in case of heterozygous DI genotype (Fig. 1). All samples that were identified initially as a DD genotype were reanalyzed using an insertion-specific primer pair, as reported by Lindpaintner et al [30], except that the annealing temperature was 67°C. A 335 bp band was obtained only in the presence of the I allele and no bands were detected for samples with DD genotype. PCR was carried out in a final volume of 25 µL containing 50 ng genomic DNA template, 1 X PCR buffer (Fermentas) with 2 mM MgCl_2_, 0.4 µM of each primer (Genescript), 50 µM dNTPs (Fermentas) and 0.5 U DNA polymerase (Fermentas). For PCR amplification, the stan­dard program was used as follows: one initial denaturation step at 94°C for 7 min, followed by 30 denaturation cycles of 30 s at 94°C, 45 s of annealing at 58°C, and 45 s of extension at 72°C, fol­lowed by a final elongation cycle at 72°C for 7 min.

**Table 1:** Primers for ACE gene Polymorphism

Target Codon	Sequence	Amplicon (bp)	T_m_ (°C)
ACE	F 5’-CTGGAGACCACTCCCATCCTTTCT -3’ R 5’- GATGTGGCCATCACATTCGTCAGAT -3’	490bp for II 190bp for DD	58
ACE I	F5-TGGGACCACAGCGCCCGCCACTAC -3’ R5- TCGCCAGCCCTCCCATGCCCATAA-3’	335bp	67

DNA amplicons were electrophoresed through a 2-3% agarose gel for resolution. The genotypes of more than 20% of the samples were double blindly reassessed to confirm the results by two independent researchers. A positive control for each polymorphism was used for 50% of samples.

### Statistical analysis

Observed frequencies of genotypes in hypertensive patients were compared to con­trols using chi-square or Fisher exact tests when expected frequencies were small. The chi-square test was used to verify whether genotype distributions were in Hardy-Weinberg equilibrium. Statistical significance was set at P < 0.05. Statistical analyses were performed with PASW version 18 Software.

## Results

A total of 52 hypertensive patients and 150 control subjects were included in this study. Subjects with completely normal echo-cardiographic study were taken as controls. The patients comprised 38 males and 14 females (M/F ratio = 2.7) and the control subjects consisted of 100 males and 50 females (M/F ratio = 2). Mean age in patients and control groups was 52 years. No significant gender- or age-related differences were ob­served between the groups (P > 0.05). Furthermore, among hypertensive patients 30 were rural and 22 urban; 22 were smokers and 30 non smokers (Table 2).

**Table 2:** Frequency Distribution Analysis of Selected Demographic and Risk Factors in Hypertensive Cases and Controls

Variable	Cases (n = 52)	Controls (n = 150)	P Value*
Age group			
< 50	25	77	0.74
≥ 50	27	73	
Gender			
Female	14	50	0.48
Male	38	100	
Dwelling			
Rural	30	78	0.52
Urban	22	72	
Smoking status			
Never	30	92	0.74
Ever	22	58	

*Two tailed fisher test

In this study, among 52 hypertensive cases we found the frequency of ACE DD genotype to be 46.15% (24/52), II 23.07% (12/52) and DI 30.77% (16/52), while as in general control (150) population the DD frequency is 53.33% (80/150), II 14.00% (21/150) and DI 32.77% (49/150). The association of ACE I/D polymorphism with the hypertensive cases was not found to be significant (p > 0.05) (Table 3).

**Table 3:** Genotype Frequencies of ACE Intron 16 Gene Polymorphism in Cases and Controls

ACEs Genotype	Cases (n = 52)	Controls (n = 150)	P Value
DD	24 (46.15 %)	80 (53.33%)	
DI	16 (30.77%)	49 (32.77%)	0.245367
II	12 (23.07%)	21 (14.00%)	

Furthermore, the hazard ratio of ACE DI genotype in hypertensive cases was found to be 1.08 times and of ACE II genotype 1.90 times that of general control population, indicating thereby that ACE I allele is associated with the increased risk of hypertension (Table 4).

**Table 4:** Genotype Frequencies of ACE Intron 16 Gene Polymorphism in Cases and Controls

ACEs Genotype	Cases (n = 52)	Controls (n = 150)	OR (95% CI )
DD	24 (46.15 %)	80 (53.33%)	Ref (1)
DI	16 (30.77%)	49 (32.77%)	1.08 (0.52 - 2.24)
II	12 (23.07%)	21 (14.00%)	1.90 (0.81 - 4.42)
DI+II	28 (53.84%)	69 (46.77%)	1.35 (0.71 - 2.54)

Echo-cardiographic analysis of hypertensive cases revealed that nine cases had mild, 27 had moderate and 16 had severe calcification (Table 5 and 6). ACE I/D analysis showed that among 27 moderate AVC cases, eight had DI and two II genotype; and among 16 severe AVC cases, five had DI and eight II genotype. Also, 53.5% (23/43) higher AVC cases had ACE I allele in either homozygous or heterozygous form.

**Table 5:** Relationship of ACE I/D Polymorphism and AVC Status

ACE Genotype	AVC Status		
	Mild (n = 9)	Moderate (n = 27)	Severe (n = 16)
DD (n = 24)	4	17	3
DI (n = 16)	3	8	5
II (n = 12)	2	2	8
DI + II (n = 28)	5	10	13

**Table 6:** Correlation of ACE I/D Polymorphism and AVC Status

ACE Genotype	AVC Status		
	Mild (n = 9)	Moderate/Severe (n = 43)	OR (95% CI)
DD (n = 24)	4	20	Ref = 1
DI (n = 16)	3	13	1.15 (0.22 - 6.02)
II (n = 12)	2	10	1.0 (0.16 - 6.42)
DI + II (n = 28)	5	23	1.08 (0.26 - 4.6)

## Discussion

Kashmir also called ‘Pir-e-Waer’ is one of the picturesque place on earth located in the northern part of India. In between, the Himalayas is home of one of the oldest ethnic population that has been proven beyond doubt to be exposed to a special set of environmental and dietary risks which include consumption of sun-dried and smoked fish and meat, dried and pickled vegetables, red chilly, Hakh (a leafy vegetable of *Brassica* family), hot noon chai (salted tea), and Hukka (water pipe) smoke [31, 32].

The etiology of primary hypertension is unknown however its diverse hemodynamic and pathophysiologic derangements are unlikely to result from a single cause [33]. Heredity is one of the main predisposing factors, but the exact mechanism is unclear [34]. Various environmental factors (for exemple, high salt intake, obesity, stress) seem to act only in genetically susceptible persons [35]. The renin-angiotensin system (RAS) has been identified by many studies to be the most important of the endocrine systems that affect the control of blood pressure [36-38].

In our study on hypertensive patients of Kashmir, we found that among 52 hypertensive cases the frequency of ACE DD genotype to be 46.15% (24/52), II 23.07% (12/52) and DI 30.77% (16/52) however no statistical significance was observed between the ACE gene I/D polymorphism and hypertension. This was in tune with other studies [38-40].

However, on the other hand, a significant association of the ACE D allele with hypertension in African Americans, Chinese and Japanese population has already been established [41-44]. In one of the seminal study in the same region of the sub-continent in Pakistani population, it was shown that I allele is associated with hypertension [45]. The association of I allele with hypertension in Pakistani population may be attributed to the presence of high levels of inbreeding, thereby resulting in higher heterozygosity. The heterogeneity in association of ACE I/D polymorphism with essential hypertension may be either due to varied ethnicity [21] or the various other genetic and environmental factors implicated in the regulation of blood pressure [46]. Any variation in even a single etiological factor could lead to difference in blood pressure and thereby hypertension. Another study carried out in Bangladeshi population found a significant association of ACE I/D polymorphism with hypertension [47]. In other two studies on ACE I/D polymorphism in hypertensive cases, carried out on two geographical opposite populations (Punjabi and Southern) in India, a positive association was observed [48, 49].

Furthermore, Echo-cardiographic analysis of hypertensive cases in relation to ACE genotype revealed among 27 moderate AVC cases, eight had DI and only two had II genotype; and among 16 severe AVC cases, five had DI and eight had II genotype. Also, 53.5% (23/43) higher AVC cases had ACE I allele in either homozygous or heterozygous form. The ACE I/D polymorphism has been already identified as one of the risk factors of AVC [50]. The insertion/deletion polymorphism in the ACE gene is correlated with the circulating ACE levels. Individuals with II genotype have the lowest circulating ACE levels as compared to DD genotype, which are known to have high ACE levels [51].

In conclusion, the present study carried out for the first time in our population clearly indicates the strong association of ACE I/D polymorphism with hypertension. However, the data need validation in a large cohort study.
